# Requirement of SLD5 for Early Embryogenesis

**DOI:** 10.1371/journal.pone.0078961

**Published:** 2013-11-11

**Authors:** Tomomi Mohri, Masaya Ueno, Yumi Nagahama, Zhi-Yuan Gong, Masahide Asano, Hiroko Oshima, Masanobu Oshima, Yasushi Fujio, Nobuyuki Takakura

**Affiliations:** 1 Department of Signal Transduction, Research Institute for Microbial Diseases, Osaka University, Suita, Osaka, Japan; 2 Laboratory of Clinical Science and Biomedicine, Graduate School of Pharmaceutical Sciences, Osaka University, Suita, Osaka, Japan; 3 Institute for Experimental Animals, Kanazawa University Advanced Science Research Center, Kanazawa, Ishikawa, Japan; 4 Division of Genetics, Cancer Research Institute, Kanazawa University, Kanazawa, Ishikawa, Japan; 5 Japan Science and Technology Agency, Chiyoda-ku, Tokyo, Japan; Osaka University Graduate School of Medicine, Japan

## Abstract

SLD5 forms a GINS complex with PSF1, PSF2 and PSF3, which is essential for the initiation of DNA replication in lower eukaryotes. Although these components are conserved in mammals, their biological function is unclear. We show here that targeted disruption of SLD5 in mice causes a defect in cell proliferation in the inner cell mass, resulting in embryonic lethality at the peri-implantation stage, indicating that SLD5 is essential for embryogenesis. Moreover, this phenotype of SLD5 mutant mice is quite similar compared with that of PSF1 mutant mice. We have previously reported that haploinsufficiency of PSF1 resulted in failure of acute proliferation of bone marrow hematopoietic stem cells (HSCs) during reconstitution of bone marrow ablated by 5-FU treatment. Since SLD5 was highly expressed in bone marrow, we investigated its involvement in bone marrow reconstitution after bone marrow ablation as observed in PSF1 heterozygous mutant mice. However, heterozygous deletion of the SLD5 gene was found not to significantly affect bone marrow reconstitution. On the other hand, abundant SLD5 expression was observed in human cancer cell lines and heterozygous deletion of the gene attenuated tumor progression in a murine model of spontaneous gastric cancer. These indicated that requirement and dependency of SLD5 for cell proliferation is different in different cell types.

## Introduction

In mammals, most adult somatic cells are quiescent. However, organ-specific stem or progenitor cells can be induced to proliferate in response to tissue damage and subsequently give rise to tissue-specific differentiated cells. In the bone marrow, hematopoietic stem cells and progenitor populations in the vascular niche continue to cycle [1]. To identify genes involved in proliferation of stem cells or progenitor cells in the adult, we constructed a hematopoietic stem cell-specific library and cloned the mouse ortholog of PSF1 (partner of SLD5-1), and its binding partner, SLD5 [2], [3].

PSF1 and SLD5 were first identified in lower eukaryotes and were reported to form a GINS complex with PSF2 and PSF3. The GINS complex is essential for cell growth in lower eukaryotes, as it regulates both the initiation and progression of DNA replication [4]–[6]. The initiation of DNA replication is controlled by a highly ordered series of steps involving multiple complexes at replication origins [7], [8]. This commences with the binding of the six-subunit origin recognition complex (ORC) to replication origins. CDC6 and Cdt1 bind to ORCs to act as loading factors for the Mcm2-7 (minichromosome maintenance) complex followed by the establishment of a prereplication complex (pre-RC). At the G1/S transition of the cell cycle, the pre-RCs are transformed into initiation complexes. Activation of MCM helicase activity requires the action of two protein kinases, DDK (Cdc7-Dbf4) and CDK (cyclin-dependent), as well as the participation of at least eight additional factors, including Mcm10, Cdc45, Dpb11, synthetic lethal with dpb11 mutant-2 (Sld2), Sld3, and GINS [9].

The GINS complex has been suggested to play an important role in the control of cell growth in lower eukaryotes. In *Drosophila*, SLD5 is essential for normal cell cycle progression and the maintenance of genome integrity [10]. Although the genes encoding its components are evolutionarily conserved, the functions of the GINS complex in mammalian cells have not yet been elucidated. We previously reported that targeted disruption of the PSF1 gene in mice led to early embryonic lethality at embryonic day 6.5 owing to growth arrest of the inner cell mass (ICM) [2]. If the function of the GINS complex is conserved in mammalian cells, SLD5 gene ablation in mice should lead to a defective phenotype similar to that observed in PSF1 mutant embryos. On the other hand, it may be that the factors constituting the GINS complex also function individually without forming a heterotetramer. It has been demonstrated that ATM and ATR kinases phosphorylate PSF2 but not other GINS factors, suggesting that PSF2 might be related to the response to DNA damage [11]. During embryonic development in Xenopus laevis, the patterns of gene expression of GINS factors are similar, but there are differences in tissue-specific expression [12] Moreover, although PSF2 plays important roles in normal eye development in Xenopus laevis, it was suggested that it might function as a transcription factor rather than as a component of GINS in this event [13]. Therefore, it is possible that SLD5 might also function independently of the formation of the GINS complex.

In the present study, we generated SLD5 mutant mice by replacing one allele of the SLD5 gene with a cDNA encoding the LacZ gene, the product of which thus served as a surrogate marker to visualize SLD5 expression. Using this mouse, we investigated the expression and function of SLD5 in the embryo. We have previously reported that haploinsufficiency of PSF1 resulted in failure of acute proliferation of bone marrow hematopoietic stem cells (HSCs) during reconstitution of bone marrow ablated by 5-FU treatment [14]. Therefore, we investigated whether SLD5 heterozygous mice show defects similar to those of PSF1 mutant mice in the adult. Furthermore, we investigated the involvement of SLD5 in tumorigenesis using SLD5-deficient heterozygous mice in a murine model of spontaneously occurring gastric cancer.

## Materials and Methods

### Animal Experiments

C57BL/6 mice were purchased from Japan SLC (Shizuoka, Japan). All animal studies were approved by the Osaka University Animal Care and Use Committee. The bone marrow reconstitution assay was performed as previously described [14]. In brief, 8-week-old wild-type and SLD5^+/−^ mice received a single tail vein injection of 5-fluorouracil (5-FU; 150 mg/kg body weight; Kyowa Hakko Kirin, Tokyo, Japan) for bone marrow ablation. The Wnt1/C2mE transgenic mouse was used as a genetic model of spontaneous gastric carcinogenesis [15]. SLD5^+/−/^Wnt1/C2mE transgenic mice were generated by mating Wnt1/C2mE transgenic mice and SLD5^+/−^ mice.

### SLD5 Gene Targeting

We isolated genomic clones encoding SLD5 from the mouse 129Sv/J library (Stratagene, La Jolla, CA) using the murine SLD5 cDNA as a probe. In the targeting construct, the region between exon 2, which contains the first ATG, and exon 5 was replaced by the NLS-β-galactosidase gene fused to a pMC1-neomycin-resistance gene. We linearized this construct with XhoI and electroporated it into 129Sv/J E14.1 embryonic stem cells. We selected G418-resistant clones and screened them by PCR and Southern blot analysis to identify the correct recombinant. Chimeric mice were generated as described previously [2]. We mated chimeric males with C57BL/6J females and screened the offspring by Southern blot and PCR analyses for those bearing the SLD5^+/−^ genotype. Embryos were collected and cultured as described [2]. Histological analysis and BrdU incorporation assays were also performed as described [2].

### X-gal Assay

For X-gal staining, dissected embryos or tissues were fixed for 15–60 min (depending on the stage) with 4% paraformaldehyde (PFA) in PBS on ice. Embryos were rinsed twice with PBS, then stained with X-Gal (1 mg/ml, Wako Fine Chemicals, Osaka, Japan) in 2 mM MgCl_2_, 0.02% NP-40, 0.1 M PBS (pH 7.5), 5 mM K_4_Fe(CN)_6_, and 5 mM K_3_Fe(CN)_6_ at 37°C for 4–16 h with shaking. Embryos were rinsed in PBS and postfixed overnight in 4% PFA.

### Immunohistochemical Analysis

Immunohistochemical analyses were carried out as previously described [16]. Tibiae and femurs were harvested from C57BL/6 mice, fixed with 4% PFA in PBS and decalcified with EDTA solution. Fixed tibiae and femurs were embedded in polyester wax (VWR international, Lutterworth, UK) and sectioned at 10-µm thickness. Sections were heated in citrate buffer to expose the antigen and then incubated with rat anti-SLD5 antibody (GeneStem, Osaka, Japan) as the primary antibody and horseradish peroxidase-conjugated goat anti-rat IgG as the secondary antibody. For visualization of HRP, 3,3′-diaminobenzidine solution was used. Images of stained sections were captured by digital microscopy (DM5500B; Leica Microsystems, Wetzlar, Germany).

### Preparation of Bone Marrow Cells and Flow Cytometry

Preparation of bone marrow cells and flow cytometric analysis were performed as previously described [14], [17]. In brief, bone marrow cells were collected from femora and tibiae and red blood cells were depleted by the standard method. Cells were incubated with anti-CD16/32 antibody to block Fc receptors and then stained with fluorescence- or biotin-conjugated antibodies. Antibodies used in flow cytometric analysis were FITC-conjugated anti-CD34 (BD Pharmingen, San Jose, CA), FITC- or PE-conjugated anti-lineage markers (CD11b, Gr-1, B220, TER119, CD4 and CD8; all purchased from eBioscience, San Diego, CA), APC-conjugated anti-c-Kit (BD Pharmingen) and/or biotinylated anti-Sca-1 antibody (BD Pharmingen). Biotinylated anti-Sca-1 antibody was visualized with PerCP-conjugated Streptavidin. Flow cytometric analysis was performed on a FACS AriaII and FACS Calibur (BD Biosciences, San Jose, CA). Data were analyzed with FlowJo software. Cell sorting of HSCs was performed using the FACS AriaII.

### Quantitative RT-PCR

Total RNA was isolated from murine tissues and cells using the RNeasy Plus Mini Kit (Qiagen, Chatsworth, CA), according to the manufacturer’s protocol. Total RNAs from human liver and lung were purchased from Clontech (Mountain View, CA). Total RNAs were reverse transcribed using the PrimeScript RT reagent Kit (Takara, Osaka, Japan). Quantitative RT-PCR (qRT-PCR) was performed using SYBR Premix Ex Taq II (Takara) on an Mx3000 (Stratagene). Levels of the specific amplified genes were normalized to the level of GAPDH. We used the following primer sets: 5′-GAT CCG CTA TGT CCT CAG CAG C-3′ and 5′-GTG TGG TCC ATA TAC TCT TTG-3′ (for mouse SLD5); 5′-CAT CAC CAT CTT CCA GGA GCG-3′ and 5′-GAG GGG CCA TCC ACA GTC TTC-3′ (for mouse GAPDH), 5′-TCC GCT ACG TCC TCA GCA GC-3′ and 5′-GTG TTC GCC ATG AAC TCT CTG-3′ (for human SLD5); 5′-GAA GGT GAA GGT CGG AGT C-3′ and 5′-GAA GAT GGT GAT GGG ATT TC-3′ (for human GAPDH).

### Cell Culture

Gastric cancer cell lines (AZ521 and HGC27), colon cancer cell lines (Colo320DM, HCT116 and SW837), a breast cancer cell line (MCF7), and a prostate cancer cell line (PC3) were kindly provided by Dr. Yutsudo (Osaka University, Japan). HeLa and HEK293T cells were obtained from the American Type Culture Collection and maintained in DMEM supplemented with 10% fetal calf serum, penicillin (100 units/ml), and streptomycin (100 µg/ml), in a humidified atmosphere containing 5% CO2 at 37°C.

### Statistical Analysis

Results are expressed as the mean ± standard deviation (SD) or the mean ± standard error (SE). Student’s *t*-test was used for statistical analysis. Differences were considered statistically significant if the *P*-value was less than 0.05.

## Results

### SLD5 is Essential for Early Embryogenesis

**Figure 1:**
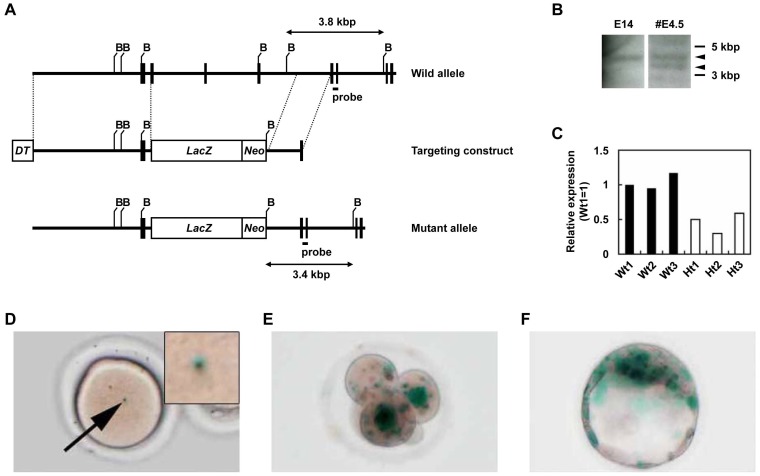
Targeted disruption of murine SLD5 and expression of SLD5 during early embryogenesis. (A) Generation of SLD5-deficient mice. Exons of SLD5 were replaced by homologous recombination with a β-galactosidase-encoding gene (lacZ) fused to a neomycin resistance gene driven by the MC1 promoter (Neo). This targeted mutation decreased the size of the BamHI restriction fragment from 3.8 kb to 3.4 kb. Exons are represented by vertical bars and introns by intervening horizontal lines. B, BamHI. (B) Southern blot analysis of wild-type (E14) and targeted (#4.5) embryonic stem cells demonstrated homologous recombination at the SLD5 locus. Arrow heads indicate wild-type (upper) and mutant (lower) genes, respectively. (C) qRT-PCR analysis of SLD5 mRNA expression in the testis from wild-type (Wt1, 2, and 3) and heterozygous SLD5 mutants (Ht1, 2, and 3). The values are normalized to the amount of mRNA in Wt1. (D-F) SLD5 promoter activity was monitored by X-gal staining, which detects β-galactosidase expression from the targeted SLD5 allele. Mature oocyte derived from super-ovulated SLD5^+/−^ female mice (D). Four-cell embryo (E) and blastocyst (F) derived by intercrosses between SLD5^+/−^ male and wild-type female mice.

As a first step towards a better understanding of the physiological role of SLD5 in mammals, we generated SDL5-deficient mice. The SLD5 gene was disrupted by replacement of the region between exons 2 (containing the start codon) and 5 with a fusion gene comprising the coding regions of LacZ and neo using standard gene targeting techniques (**Figure 1A**). Among the 22 independent embryonic stem cell colonies examined, we found one homologous recombinant (#4.5). The correct targeting of the SLD5 locus was confirmed by Southern blot analysis using a 3′ probe external to the targeting vector (**Figure 1B**). The embryonic stem cell clone, #4.5, was aggregated with C57BL/6 blastocysts to generate a chimera, which subsequently resulted in germ line transmission. The SLD5^+/−^ line was established by backcrossing with C57BL/6 mice. To confirm the loss of the SLD5 transcript in gene-disrupted mutant mice, we performed qRT-PCR on RNA from testis. As expected, the expression of SLD5 mRNA was reduced by 50% in testes of SLD5^+/−^ relative to wild-type mice with some exceptions in which SLD5 expression in SLD5+/− mice are less than 50% compared with that in wild-type mice (**Figure 1C**). SLD5^+/−^ mice were born at Mendelian frequency, and the SLD5^+/−^ heterozygotes appeared phenotypically normal and generally healthy and were fertile at up to 6 months of age.

To analyze SLD5 expression in the embryo, we took advantage of the LacZ gene cassette expressing mRNA encoding β-galactosidase under the transcriptional control of the endogenous SLD5 promoter. X-Gal staining therefore served as a reporter to monitor activity of the SLD5 promoter and SLD5 mRNA expression. Unfertilized oocytes from female SLD5^+/−^ mice stained X-Gal-positive (**Figure 1D**), suggesting that SLD5 transcripts (and possibly the maternal SLD5 proteins) were present in mature oocytes. Transcription of SLD5 was observed at the 4-cell stage when wild-type female mice were crossed with SLD5^+/−^ males (**Figure 1E**). In blastocysts, a strong X-Gal signal was observed in the ICM (**Figure 1F**). During early embryogenesis, the X-gal signal was observed specifically in intra-embryonic tissues but not in extra-embryonic tissues at E7.5. However, the signal gradually disappeared from embryonic tissues during later embryonic stages (data not shown).

**Figure 2:**
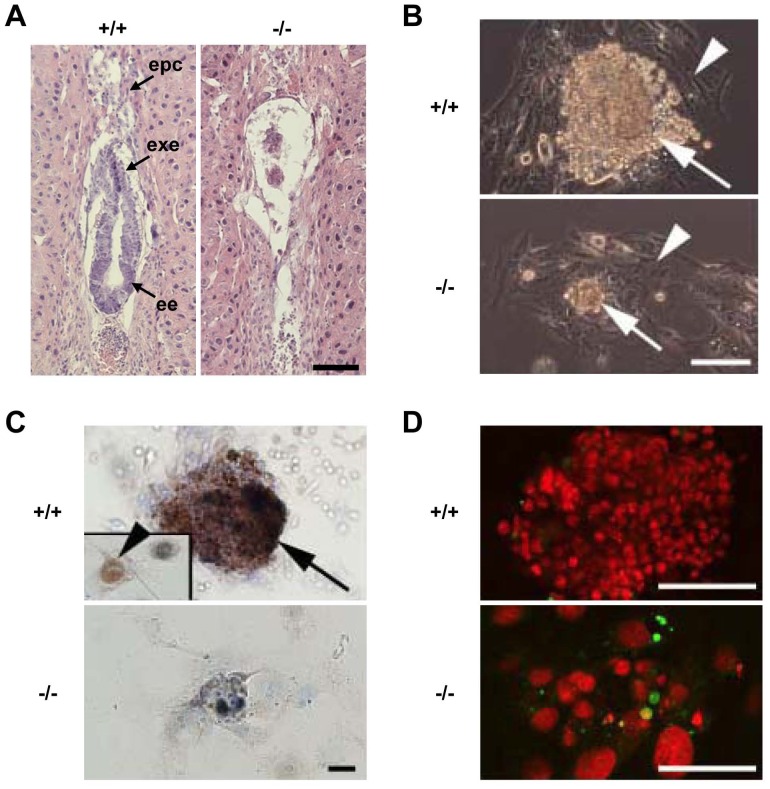
SLD5 is essential for embryogenesis. (A) Histological analysis of wild-type (+/+) and SLD5^−/−^ (−/−) embryos. Hematoxylin-eosin-stained, longitudinal sections of E6.5 embryos in decidua. epc, ectoplacental cone; exe, extraembryonic ectoderm; ee, embryonic endoderm. Scale bar, 100 µm. (B) Blastocysts (E3.5) derived from heterozygous intercrosses were cultured for 5 days, and genotyped by PCR. Representative images from cultures of wild-type (+/+) and SLD5^−/−^ (−/−) blastocysts are shown. Arrows, ICM; arrowheads, trophoblast. Scale bar, 100 µm. (C) BrdU incorporation analysis in blastocyst culture. Immunostaining with anti-BrdU antibody was performed (brown). Inset in upper panel shows a different field in this culture plate. Arrow, ICM; arrowhead, labeled trophoblast. Note that BrdU incorporation was not observed in cultures derived from SLD5-null blastocysts. Scale bar, 50 µm. (D) Apoptosis of cells in cultured ICM from blastocysts determined by TUNEL staining (green, apoptotic cells). Nuclei were counterstained with propidium iodide (red). The large nuclei observed in the lower panel are from trophoblast. Scale bar, 100 µm.

To determine the function of SLD5 in vivo, SLD5^+/−^ mice were intercrossed to produce SLD5^−/−^ mice. However, no SLD5^−/−^ neonates were obtained (**Table 1**). We next isolated and genotyped embryos at different stages and found SLD5^−/−^ blastocysts at E3.5 (**Table 1**). In normal E6.5 embryos, a cylinder-like two-layered tissue structure was observed in histological studies. However SLD5^−/−^ embryos lacked such structures (**Figure 2A**). These results indicated that SLD5^−/−^ embryos survived to the blastocyst stage and underwent implantation; however, mutants elicited hemorrhage in the decidua and subsequently died soon after implantation.

**Table 1:** Progeny of SLD5 Heterozygotes.

	No. of offspring of each genotype				
Age	+/+	+/−	−/−	No. Resolved	No. Total
E3.5	5	13	6	NA^b^	24
E5.5	ND^a^	ND	ND	4	17
E6.5	ND	ND	ND	7	27
E9.5	7	21	0	14	42
Neonate	3	7	0	0	10

a ND, not determined. The genotype of 17 E5.5 and 27 E6.5 embryos was not determined.

b NA, not available. The table shows the number of offspring obtained by mating SLD5 heterozygotes.

To further examine the SLD5^−/−^ phenotype, blastocysts from SLD5^+/−^ crosses were cultured in vitro to allow outgrowth for 5 days (**Figure 2B**). Both SLD5^+/+^ and SLD5^−/−^ blastocysts hatched from the zona pellucida and attached to culture dishes during 2 days of culture (data not shown). The development of trophoblast giant cells was found in both genotypes. However, the ICM, which forms the future embryonic tissues, failed to outgrow in SLD5^−/−^ blastocysts. According to the results of BrdU incorporation assays, vigorous DNA synthesis was occurring in SLD5^+/+^ ICM cells and trophoblasts in the first 5 days. However no BrdU incorporation was found in SLD5^−/−^ embryo**s** (**Figure 2C**). Moreover, in the ICM of SLD5^−/−^ blastocyst cultures, TUNEL-positive apoptotic cells appeared on day 4 (**Figure 2D**). Thus, the SLD5^−/−^ ICM cells were unable to proliferate and underwent apoptosis in culture. These data suggest that SLD5 is essential for cell proliferation in cells from the ICM, which is known to give rise to embryonic stem cells. Moreover, these observations were similar to those obtained using PSF1^−/−^ embryos [2], suggesting conservation of a role for the GINS complex in the control of cell growth in mammals.

### No Apparent Effect of Heterozygous Deletion of the SLD5 Gene on Reconstitution of Bone Marrow Cells after their Ablation by 5-FU

**Figure 3:**
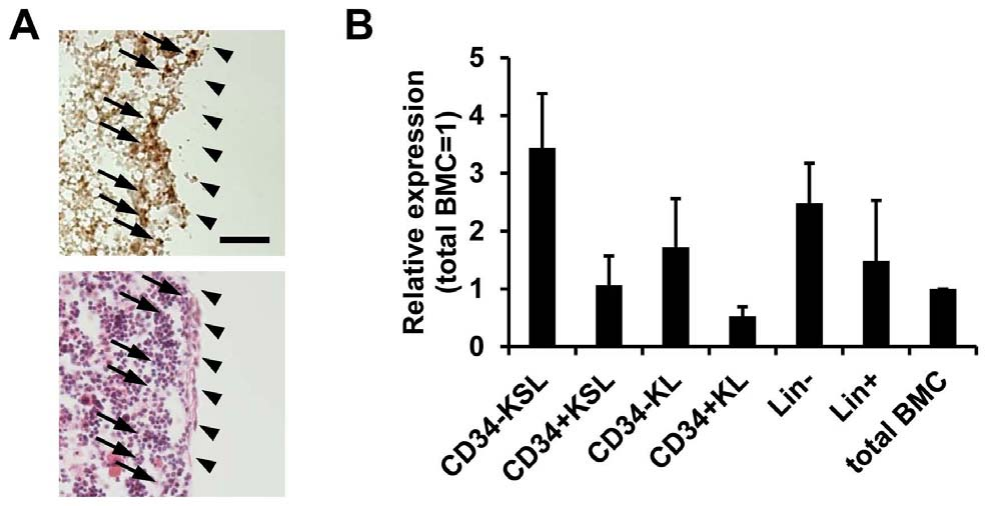
Expression of SLD5 in bone marrow cells. (A) Immunohistochemical staining for detection of SLD5 (brown; upper panel) and hematoxylin-eosin staining using serial sections (lower panel). Arrows indicate SLD5-positive cells and arrowhead**s** indicate the portion of endosteum. Scale bar, 50 µm. (B) qRT-PCR analysis of SLD5 mRNA expression in several hematopoietic cell fractions sorted from murine bone marrow cells. KSL; c-Kit^+^Sca-1^+^Lineage marker^−^, KL; c-Kit^+^Lineage marker^−^, Lin; Lineage marker, BMC; bone marrow cells. Data are shown as mean ± SE (n = 3).

We previously reported abundant expression of both SLD5 and PSF1 mRNA in bone marrow, testis and ovary, and weak expression in the thymus in adults [2], [3]. By immunohistochemical staining, SLD5-positive cells in the bone marrow seemed to localize to an area adjacent to the endosteum but not in the center of the marrow (**Figure 3A**). Because it is well-known that HSCs localize around the endosteum [18], [19] and we previously found PSF1 expression in HSCs of bone marrow [2], we analyzed SLD5 mRNA expression in several hematopoietic cell fractions sorted from murine bone marrow. We found that compared to the lineage marker-positive differentiated hematopoietic cell fraction, the lineage marker-negative progenitor cell population tends to express SLD5 more strongly. However, this difference was not significant. We previously reported that within the c-Kit^+^Sca-1^+^lineage marker^−^ (KSL) population, CD34^+^KSL, a proliferating HSC population, expresses PSF1 more strongly than CD34^−^KSL, a dormant HSC population [14]. Although the expression of SLD5 was observed in both CD34^−^KSL and CD34^+^KSL cells, the latter did not to express it as strongly as the former (**Figure 3B**). This suggests that SLD5 might be involved in hematopoiesis in a manner different from PSF1.

**Figure 4:**
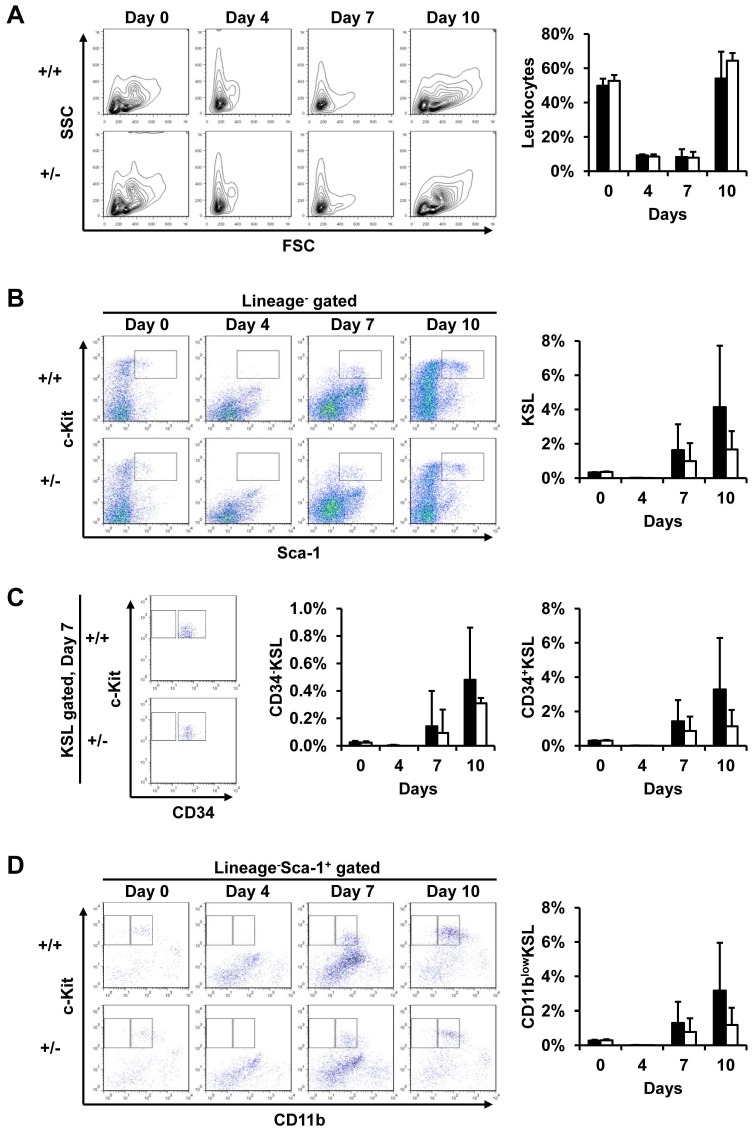
Heterozygous deletion of the SLD5 gene does not affect bone marrow cell reconstitution after their ablation by 5-FU. 5-FU was injected into 8-week-old wild-type (+/+) and SLD5^+/−^ (+/−) mice and bone marrow cells were collected thereafter. (A) Kinetics of total bone marrow cell changes after 5-FU injection. Representative data from flow cytometric analysis (left) and quantitative evaluation of frequency in the leukocyte population (right) are shown. (B-D) Time course of the frequency of HSCs during bone marrow reconstitution after 5-FU injection. (B) Representative data of flow cytometric analysis (left) and quantitative evaluation of the frequency of KSL cells (right) gated as shown in the box in the left panels are shown. (C) Flow cytometric analysis of CD34 and c-Kit expression in KSL cells on day 7 after 5-FU injection (left). The frequency of CD34^+^ or CD34^−^ KSL cells was quantitatively evaluated (right two graphs). (D) Flow cytometric analysis of CD11b and c-Kit expression in Lineage marker^−^Sca-1^+^ cells after 5-FU injection (left). The frequencies of CD11b^low^KSL are quantitatively evaluated (right). Data are represented as mean ± SD (n = 3–7 mice in each group). Notably, no significant differences in the frequency between wild-type and SLD5^+/−^ mice were found at any time point.

Based on immunohistological and PCR analysis, we hypothesized that dormant HSCs localizing at the endosteum of long bone express SLD5 relatively strongly; however, proliferating CD34^+^KSL cells also express SLD5. We previously reported that heterozygous deletion of *PSF1* gene leads to failure of acute proliferation of HSCs, resulting in severe leukocytopenia and lethality. Therefore, here we tested whether SLD5 is also required for reconstitution in a bone marrow ablation model using 5-FU [14]. However, in contrast to PSF1^+/−^ mice, SLD5^+/−^ mice survived a single injection of 5-FU (150 mg/kg body weight). The frequency and absolute numbers of leukocytes in bone marrow cells were similar in wild-type and SLD5^+/−^ mice before and after 5-FU injection (**Figure 4A**). Moreover, there are no large differences in lineage specific cell population, i.e., myeloid cells, lymphocytes, and erythrocytes between wild-type and SLD5+/− mice (data not shown). In the steady state, the frequencies of both CD34^−^ dormant and CD34^+^ proliferative KSL cells were not different in wild-type and SLD5^+/−^ mice (**Figure 4B, C**). On day 4 after 5-FU injection, the CD34^+^ and CD34^−^ KSL cells disappeared in both wild-type and SLD5^+/−^ mice (**Figure 4B**). The frequency and absolute numbers of KSL cells also gradually increased from 7 to 10 days after 5-FU injection in both. Recovery of KSL cells seemed to occur faster in both CD34^+^ and CD34^−^ KSL cells of wild-type mice; however, the difference was again not statistically significant (**Figure 4B, C**). The cycling HSC population can also be detected in CD11b^low^ cells among KSL cells [14]. We confirmed that recovery of these CD11b^low^ cells tended to be delayed in SLD5^+/−^ mice, but this difference was also not significant (**Figure 4D**). Therefore, we conclude that recovery of HSCs may be slightly delayed in SLD5^+/−^ mice compared with wild-type mice, but unlike PSF1^+/−^ animals this does not cause severe leukocytopenia and lethality.

### Suppression of SLD5 Expression Blocks Tumor Progression in a Gastric Cancer Model

**Figure 5:**
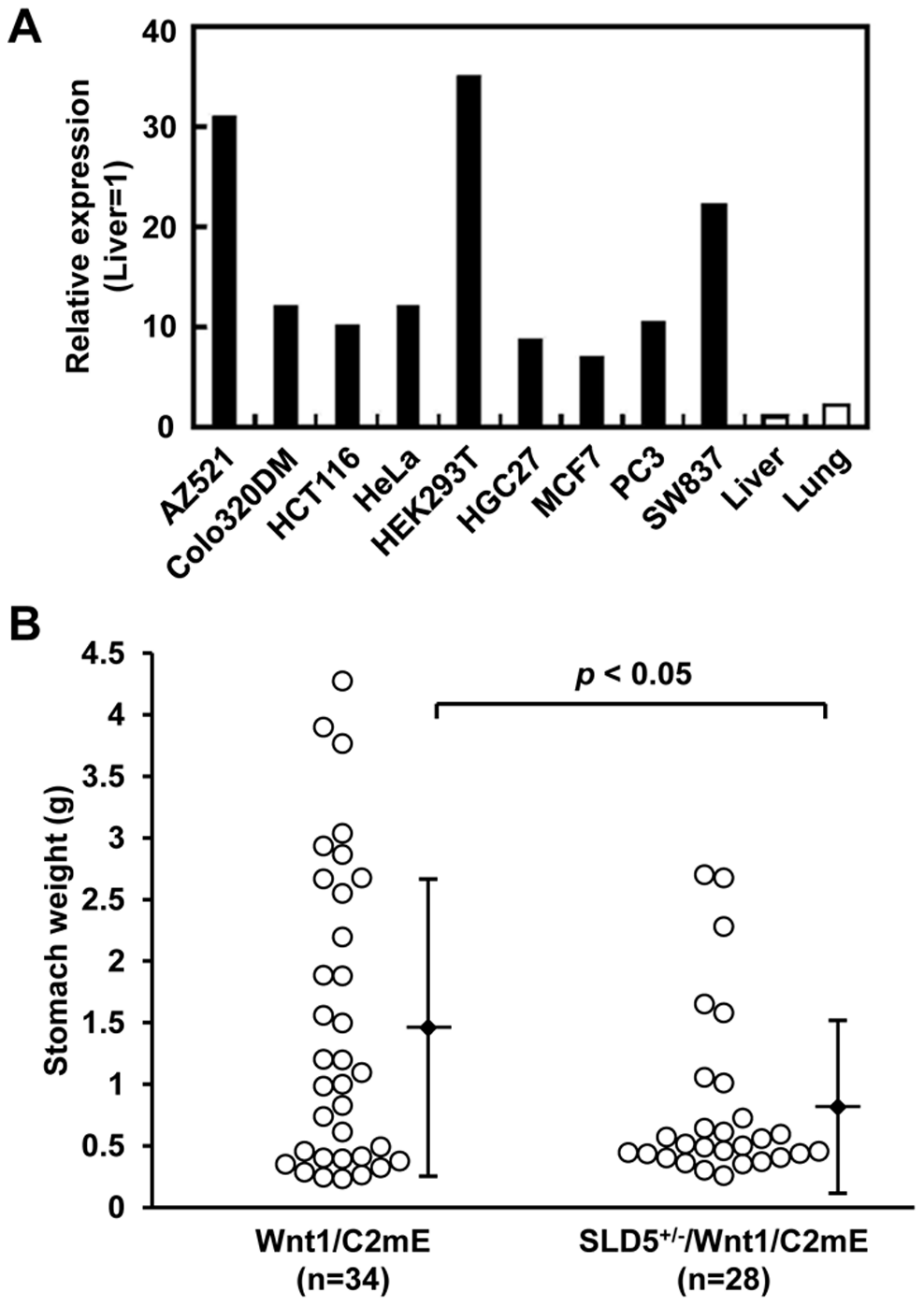
Heterozygous deletion of SLD5 gene attenuates tumor progression in gastric cancer model. (A) qRT-PCR analysis of SLD5 expression in human cancer cells and normal tissues (liver and lung). AZ521 and HGC27; gastric cancer cell lines, Colo320DM, HCT116 and SW837; colon cancer cell lines, HeLa; cervical cancer cell line, MCF7; breast cancer cell line, PC3; prostate cancer cell line. (B) The stomachs were collected from Wnt1/C2mE and SLD5+/−/Wnt1/C2mE mice at 45 weeks of age and weight of stomach including tumor was measured. Circles and horizontal bars indicate the stomach weights of each mouse and mean ± SD, respectively.

We found that the expression of SLD5 in all human cancer cell lines examined was 3-16-fold greater than in normal lung (**Figure 5A**). This suggests a requirement for SLD5 for tumor progression. To elucidate the relationship of SLD5 to cancer cell growth in tumor progression in vivo, we investigated oncogenesis in the Wnt1/C2mE transgenic mouse, a genetic model of spontaneous gastric carcinogenesis [15], on an SLD5^+/−^ background (SLD5^+/−/^Wnt1/C2mE). We found no significant differences in body weights between Wnt1/C2mE and SLD5^+/−/^Wnt1/C2mE mice at 45 weeks of age (data not shown). Although all SLD5^+/−/^Wnt1/C2mE as well as Wnt1/C2mE mice developed gastric cancer, the weights of the stomachs, including the tumor, in SLD5^+/−/^Wnt1/C2mE mice were significantly decreased compared to Wnt1/C2mE mice at this time (**Figure 5B** **and Figure S1**). This indicates that suppression of SLD5 expression attenuated tumor progression and/or delayed tumor initiation in this gastric cancer model. We propose that SLD5 is required for cell growth in gradually progressive events such as cancer development.

## Discussion

Here, we have investigated the function of SLD5 in vivo using gene targeting technology. Our results revealed impaired proliferation of ICM and trophoblast cells in SLD5^−/−^ embryos. The GINS complex and CDC45 are involved cooperatively in the initiation of DNA replication [5], [6],[20]–[24]. In mice, the phenotype of CDC45-deficient embryos after uterine implantation [25] is similar to that of SLD5- or PSF1-null embryos. Moreover, mice deficient in CDC45 show a defect in cell proliferation in blastocyst cultures [25] as observed in the present experiments using SLD5-null blastocysts. Therefore, the molecular functions of SLD5 and CDC45 in DNA replication may be conserved in mammalian cells. Although SLD5 is essential for cell proliferation in yeast [6], no obvious morphological abnormality was found in SLD5^−/−^ embryos before implantation (data not shown). Our X-gal staining experiments (**Figure 1**) suggested that maternal SLD5 transcripts are stored in unfertilized eggs. Maternal PSF1 transcripts were also observed in unfertilized oocytes [2]. These stores of maternal SLD5 transcripts could account for the growth of SLD5^−/−^ embryos through the early developmental stages, and we conclude that the timing of SLD5^−/−^ lethality is due to the loss and/or dilution of maternal SLD5 transcripts around the implantation stage.

We previously reported abundant expression of SLD5 and PSF1 in bone marrow, testis and ovary in which stem and/or progenitor cells actively proliferate and there is constant turnover of tissue-specific cells [2], [3]. Our previous results suggested that PSF1 expression was concentrated in the stem cell population within the bone marrow and testis. Similarly, β-galactosidase staining in adult SLD5^+/−^ mice did not reveal SLD5 expression in organs other than bone marrow, testis and ovary (data not shown). However, deletion of one allele of the SLD5 gene did not affect reconstitution after bone marrow ablation by 5-FU. Moreover, in the skin-flap wound-healing model using adult SLD5^+/−^ mice, we could not detect SLD5 (β-galactosidase)-positive proliferating cells in the skin during the recovery stage (data not shown). Taken together, these data suggest that SLD5 plays a role at the stem cell level particularly in embryogenesis but that it may not be critical for the induction of proliferation of certain stem/progenitor and somatic cell populations in response to other sorts of stress, such as bone marrow reconstitution and wound healing, in adults.

PSF1 was highly expressed in proliferative HSCs rather than dormant HSCs and 5-FU injection led to lethality in PSF1^+/−^ mice, resulting from a delay in induction of HSC proliferation during bone marrow reconstitution [14]. In contrast, SLD5 was predominantly expressed in dormant HSCs rather than proliferative HSCs, suggesting that it is more likely involved in maintaining stemness rather than proliferation of stem/progenitor cells. Thus, bone marrow reconstitution after 5-FU treatment occurred successfully in SLD5^+/−^ mice. However, our findings regarding the roles of SLD5 in adults were obtained using mice in which the SLD5 gene is heterozygously deleted. Therefore, conditional gene targeting of SLD5 would be required to definitively clarify the function of SLD5 in adult tissues.

Finally, we demonstrated that heterozygous deletion of the SLD5 gene attenuated tumor progression in a murine spontaneous gastric cancer model using the Wnt1/C2mE transgenic mouse [15]. This is consistent with our findings of strong expression of SLD5 as well as PSF1 [16] in most cells in the tumor cell lines we have tested. Because SLD5 is expressed in cells in the ICM, it has been suggested that it is an embryonic stem cell-specific gene [26]. Moreover, an embryonic stem cell gene expression signature is present in poorly differentiated aggressive human tumors [27]. Taken together, these data indicate that SLD5 is utilized for aggressive tumor progression. Therefore, It is likely that SLD5 is one of candidate to detect cancer stem cell and that SLD5 would be a molecular target for anti-cancer therapy, but discriminating between SLD5 functions in cancer cells and cells from normal adult tissues is required to confirm its utility as a new target in oncology.

## Supporting Information

Figure S1 **The Wnt1/C2mE transgenic mouse was used as a genetic model of spontaneous gastric carcinogenesis** [**15**] **.** SLD5^+/−/^Wnt1/C2mE transgenic mice were generated by mating Wnt1/C2mE transgenic mice and SLD5^+/−^ mice. The stomachs were collected from Wnt1/C2mE and SLD5^+/−/^Wnt1/C2mE mice at 45 weeks of age.
(PDF)
