# Supplementary material for: Requirement of SLD5 for Early Embryogenesis
Source: PLoS One. 2013 Nov 11;8(11):e78961. doi: 10.1371/journal.pone.0078961 (PMC3823970; doi:10.1371/journal.pone.0078961)
Supplement: Figure S1 — The Wnt1/C2mE transgenic mouse was used as a genetic model of spontaneous gastric carcinogenesis [15] . SLD5+/−/Wnt1/C2mE transgenic mice were generated by mating Wnt1/C2mE transgenic mice and SLD5+/− mice. The stomachs were collected from Wnt1/C2mE and SLD5+/−/Wnt1/C2mE mice at 45 weeks of age. (PDF) [file pone.0078961.s001.pdf]

Figure S1. Gross appearance of gastric cancer.

The Wnt1/C2mE transgenic mouse was used as a genetic model of spontaneous gastric carcinogenesis [1]. SLD5$^{+/-}$/Wnt1/C2mE transgenic mice were generated by mating Wnt1/C2mE transgenic mice and SLD5$^{+/-}$ mice. The stomachs were collected from Wnt1/C2mE and SLD5$^{+/-}$/Wnt1/C2mE mice at 45 weeks of age

1. Oshima H, Matsunaga A, Fujimura T, Tsukamoto T, Taketo MM, et al. (2006) Carcinogenesis in mouse stomach by simultaneous activation of the Wnt signaling and prostaglandin E2 pathway. Gastroenterology 131: 1086-1095.

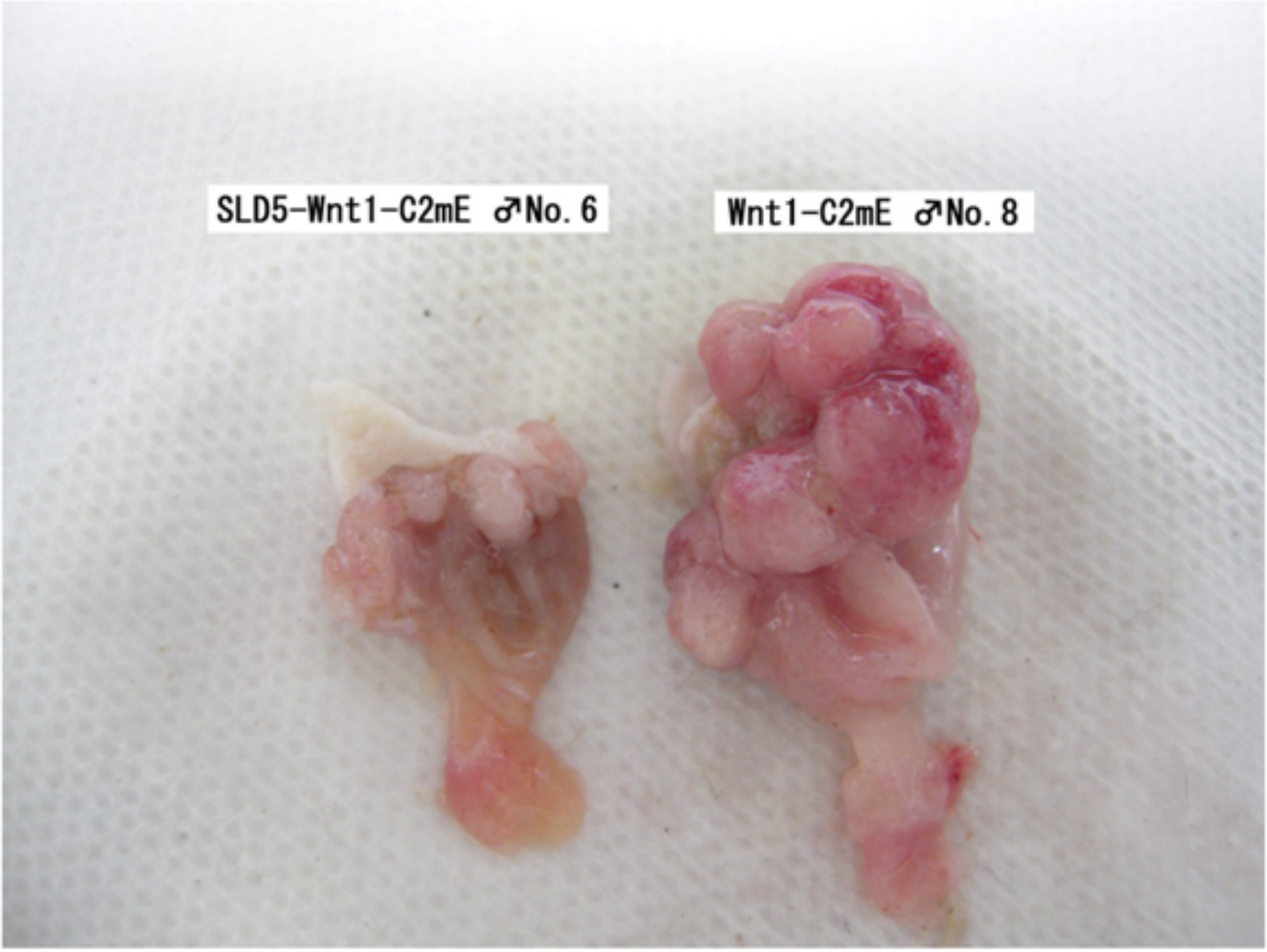

Figure S1 Mohri T et al
